# Supplementary figures and images for: Effects of rheumatoid arthritis associated transcriptional changes on osteoclast differentiation network in the synovium
Source: PeerJ. 2018 Oct 11;6:e5743. doi: 10.7717/peerj.5743 (PMC6186409; doi:10.7717/peerj.5743)

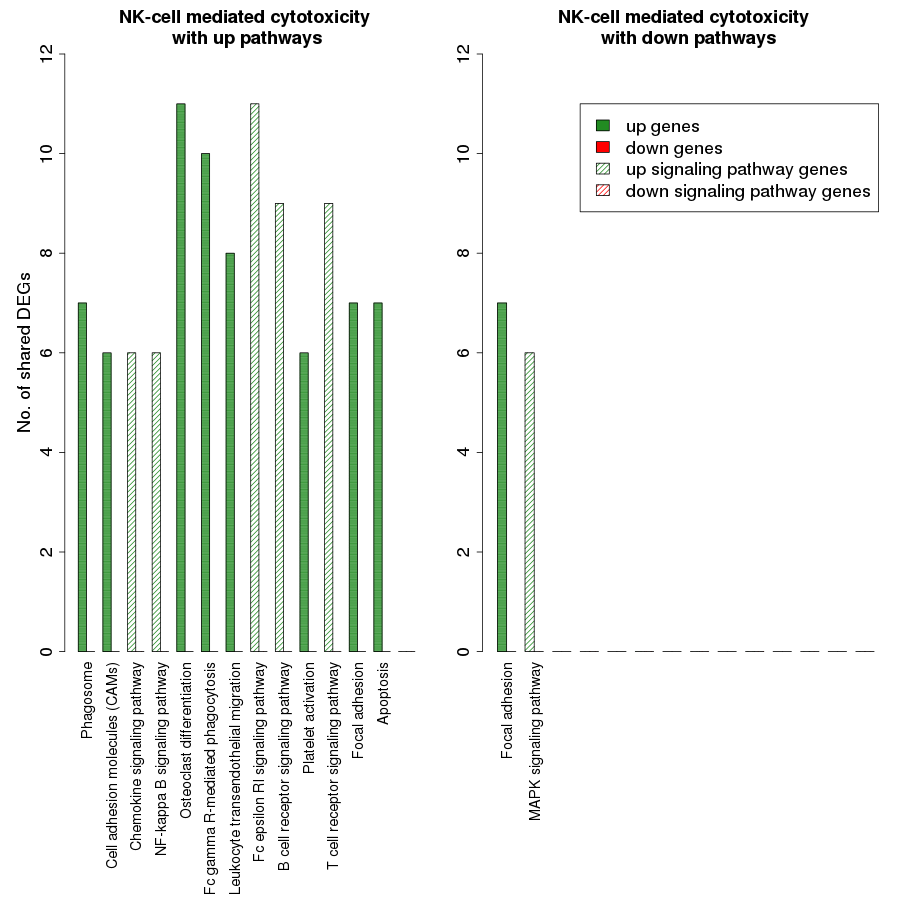

Supplement: Supplemental Information 11 — The x-axis represents the enriched non-disease pathways with which the natural killer cell mediated cytotoxicity pathway share DEGs. The signaling pathways are represented by striped bars whereas the non-signaling pathways are represented by solid bars. The y-axis denotes the number of DEGs shared between the natural killer cell mediated cytotoxicity pathway and each of the enriched non-disease pathways. [file peerj-06-5743-s011.png]

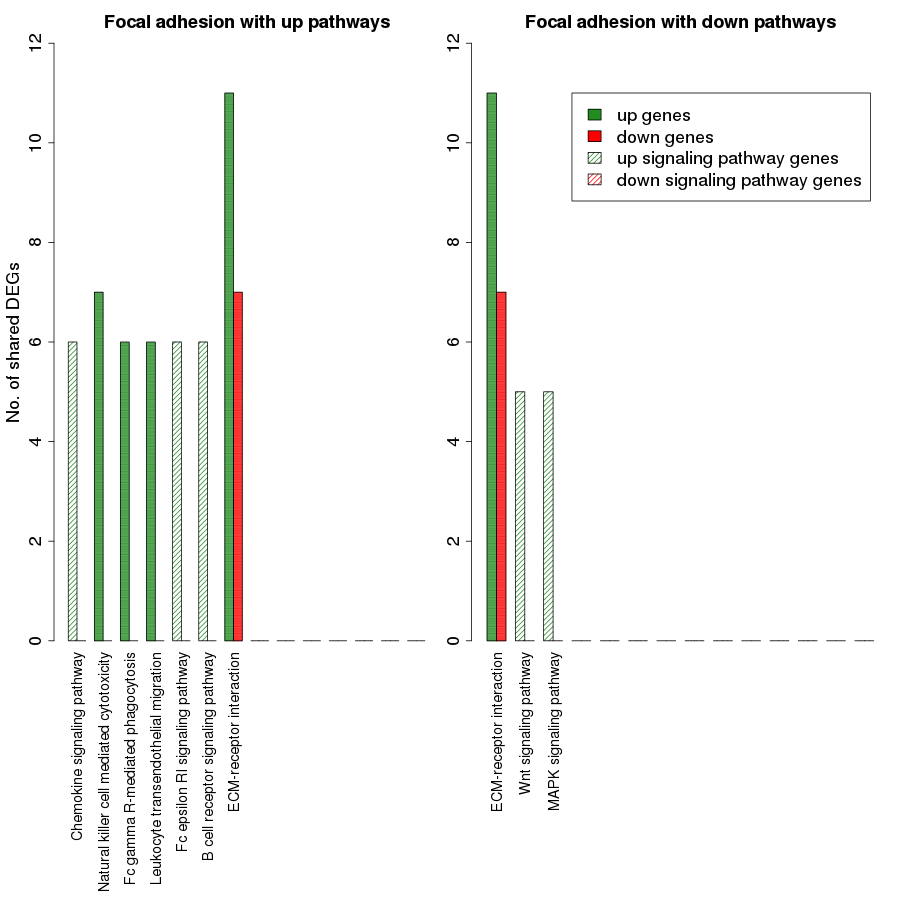

Supplement: Supplemental Information 12 — The x-axis represents the enriched non-disease pathways with which the focal adhesion pathway share DEGs. The signaling pathways are represented by striped bars whereas the non-signaling pathways are represented by solid bars. The y-axis denotes the number of DEGs shared between the focal adhesion pathway and each of the enriched non-disease pathways. [file peerj-06-5743-s012.png]

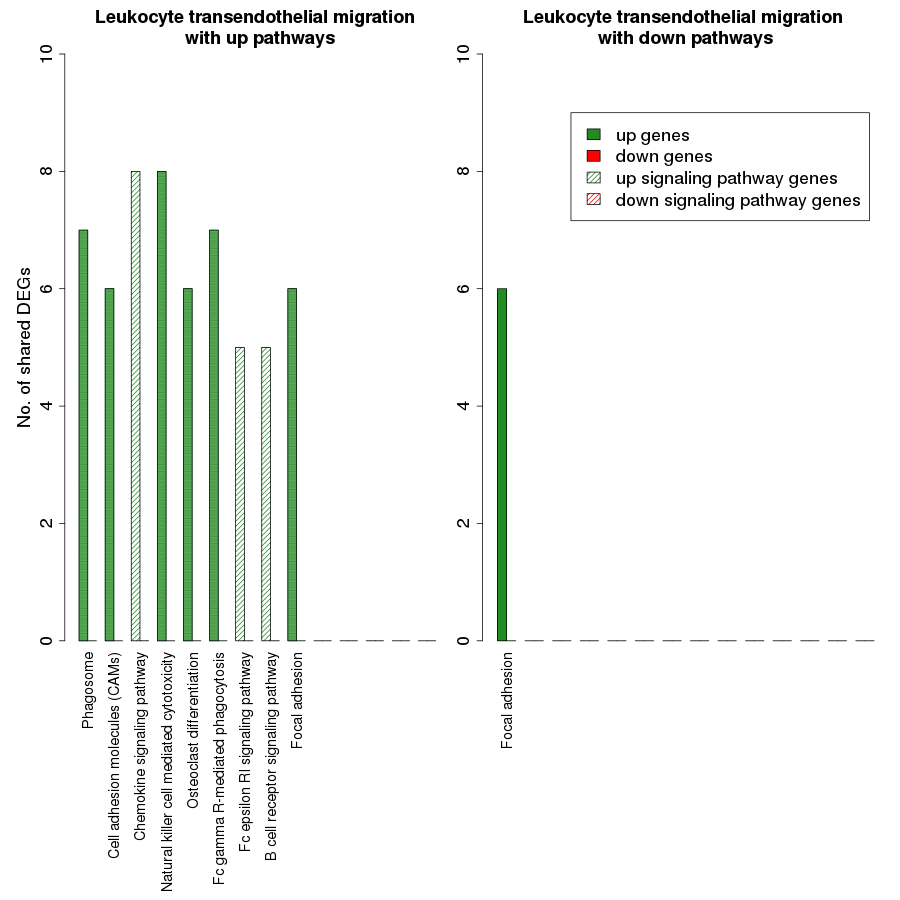

Supplement: Supplemental Information 13 — The x-axis represents the enriched non-disease pathways with which the leukocyte transendothelial migration pathway share DEGs. The signaling pathways are represented by striped bars whereas the non-signaling pathways are represented by solid bars. The y-axis denotes the number of DEGs shared between the leukocyte transendothelial migration and each of the enriched non-disease pathways. [file peerj-06-5743-s013.png]

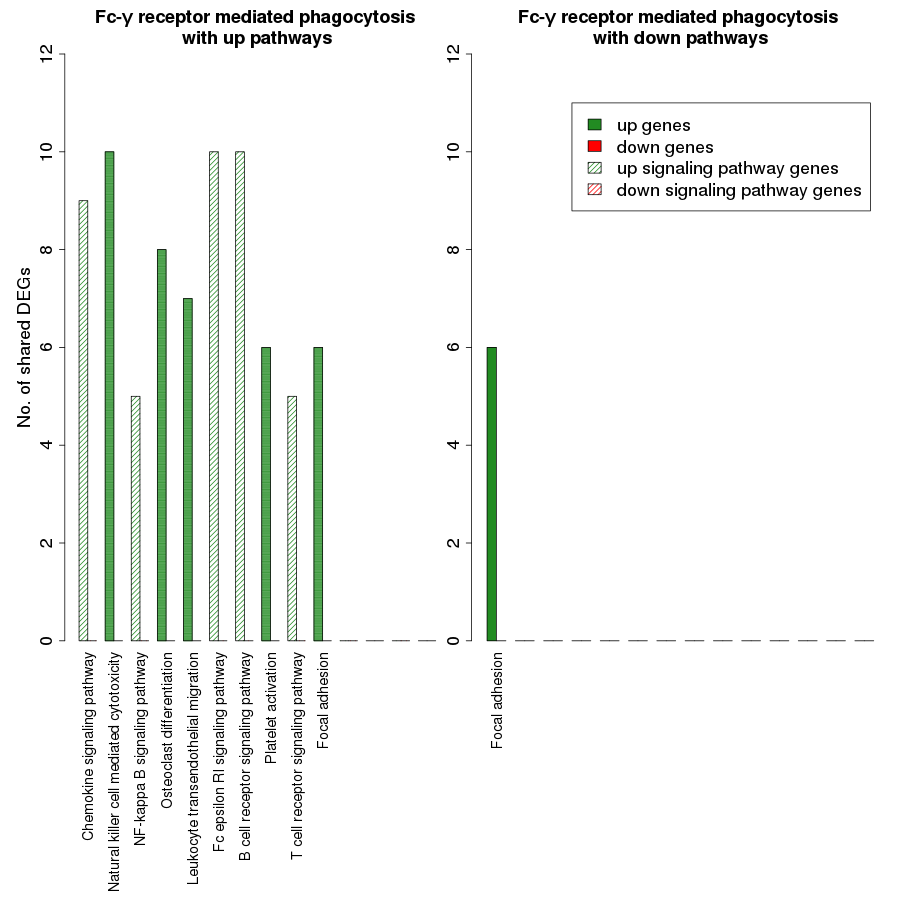

Supplement: Supplemental Information 14 — The x-axis represents the enriched non-disease pathways with which the Fc-γ receptor mediated phagocytosis pathway share DEGs. The signaling pathways are represented by striped bars whereas the non-signaling pathways are represented by solid bars. The y-axis denotes the number of DEGs shared between the Fc-γ receptor mediated phagocytosis pathway and each of the enriched non-disease pathways. [file peerj-06-5743-s014.png]

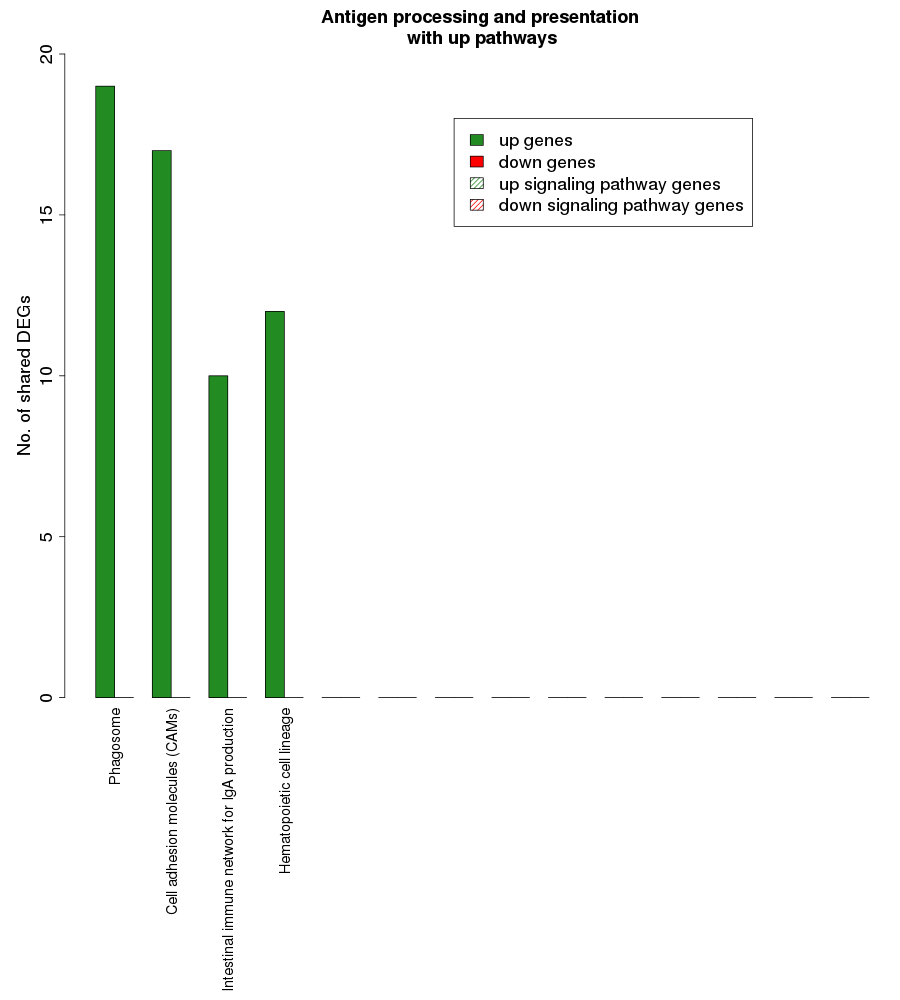

Supplement: Supplemental Information 15 — The x-axis represents the enriched non-disease pathways with which the antigen processing and presentation pathway share DEGs. The signaling pathways are represented by striped bars whereas the non-signaling pathways are represented by solid bars. The y-axis denotes the number of DEGs shared between the antigen processing and presentation pathway and each of the enriched non-disease pathways. [file peerj-06-5743-s015.png]

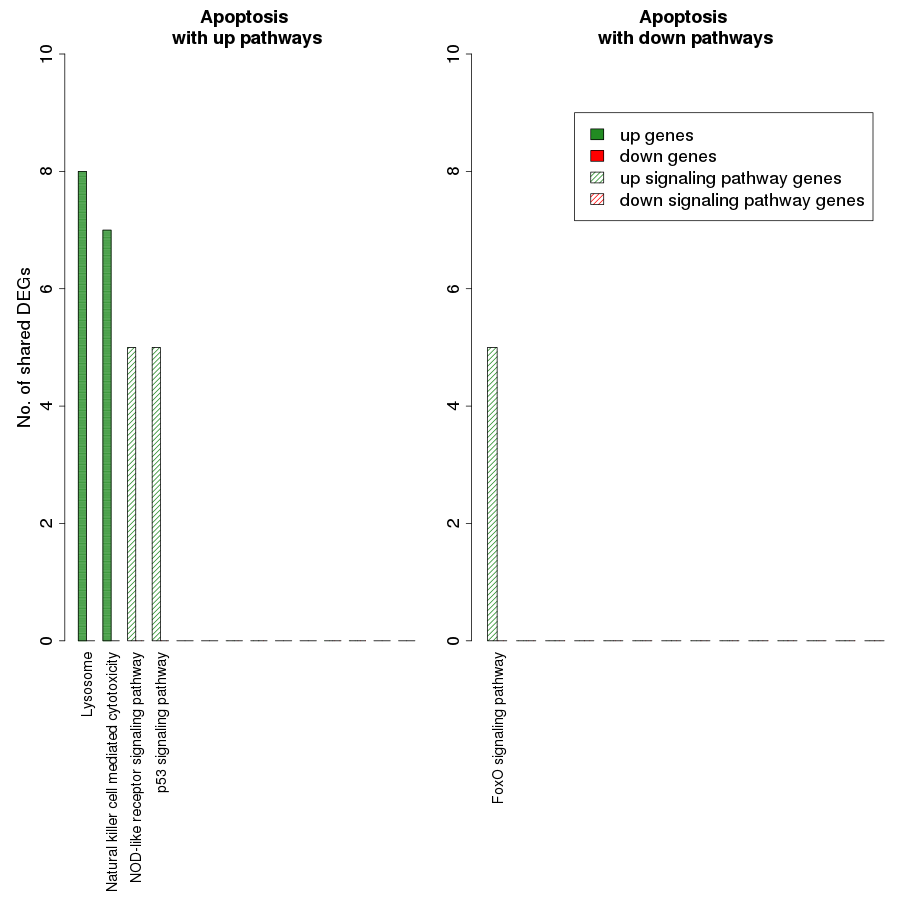

Supplement: Supplemental Information 16 — The x-axis represents the enriched non-disease pathways with which the apoptosis pathway share DEGs. The signaling pathways are represented by striped bars whereas the non-signaling pathways are represented by solid bars. The y-axis denotes the number of DEGs shared between the apoptosis pathway and each of the enriched non-disease pathways. [file peerj-06-5743-s016.png]

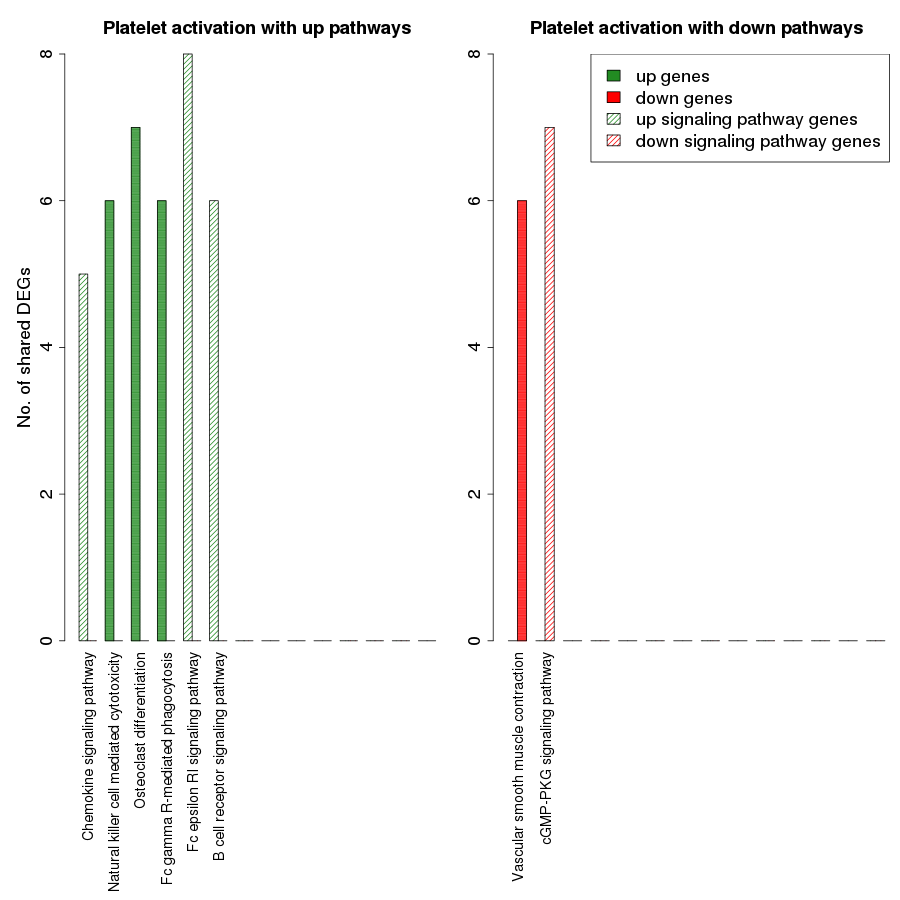

Supplement: Supplemental Information 17 — The x-axis represents the enriched non-disease pathways with which the platelet activation pathway share DEGs. The signaling pathways are represented by striped bars whereas the non-signaling pathways are represented by solid bars. The y-axis denotes the number of DEGs shared between the platelet activation pathway and each of the enriched non-disease pathways. [file peerj-06-5743-s017.png]

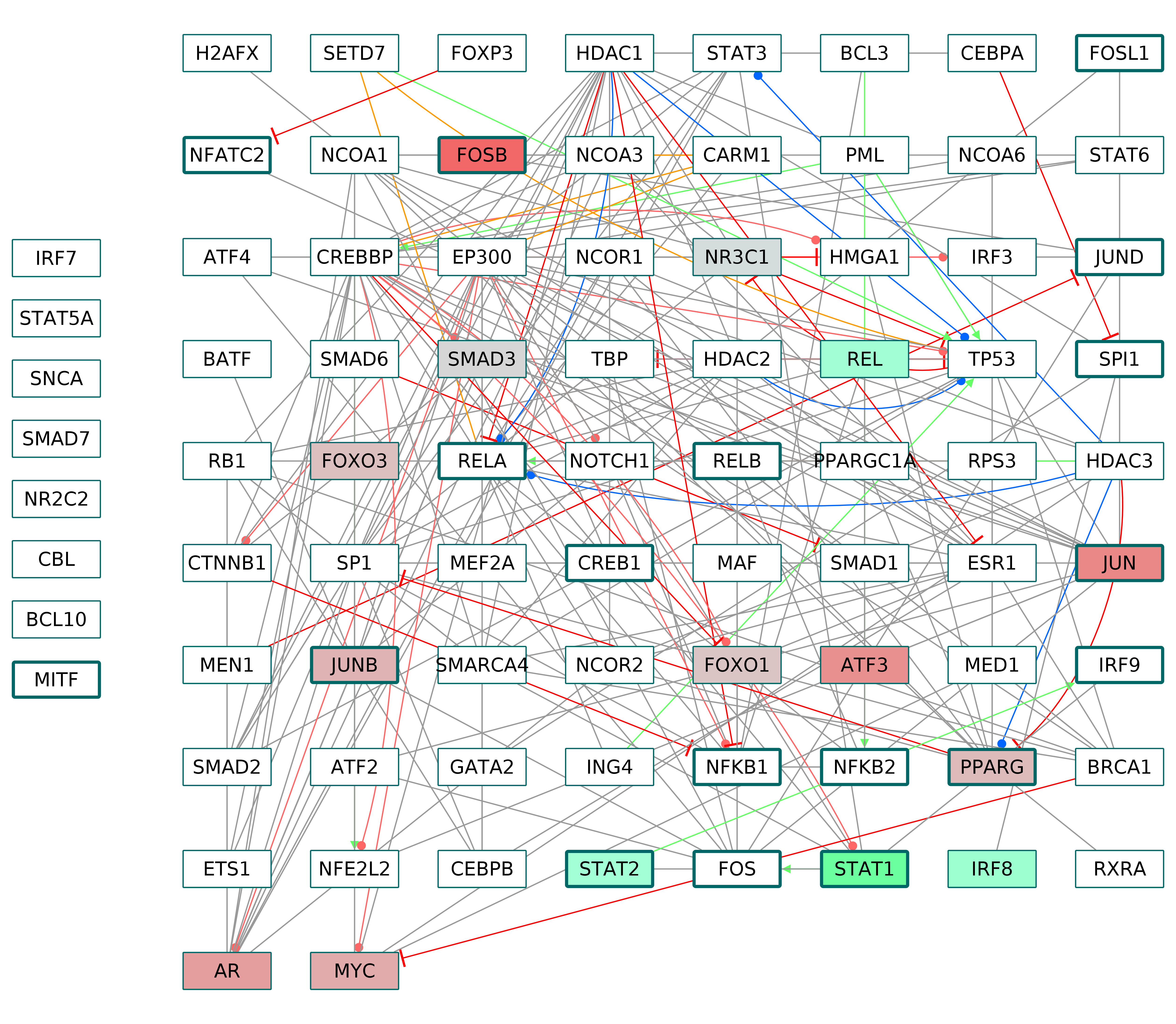

Supplement: Supplemental Information 18 — The core proteins are represented by thick borders and the shell proteins by thin borders. The RA drug targets are indicated by red borders. The degree of differential regulation of the nodes is denoted as follows: red to grey–downregulation and green–upregulation. The proteins lying isolated in the subnetwork are connected to the non-DNA-binding proteins of the directed osteoclast differentiation network. [file peerj-06-5743-s018.png]

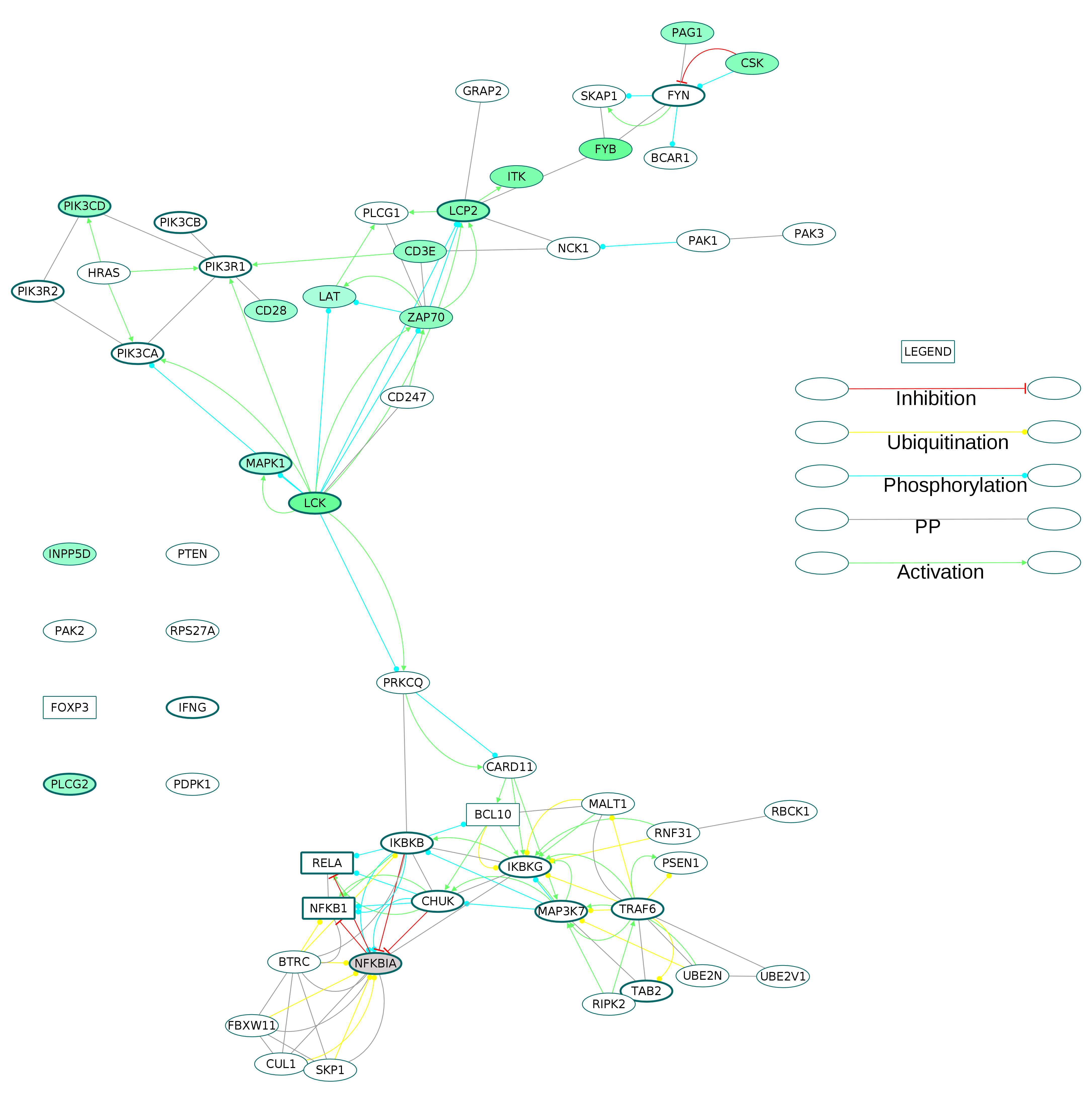

Supplement: Supplemental Information 19 — The core proteins are represented by thick borders and the shell proteins by thin borders. Rectangle nodes represent the DNA-binding proteins. The RA drug targets are indicated by red borders. The degree of differential regulation of the nodes is denoted as follows: red to grey–downregulation and green–upregulation. The proteins lying isolated in the subnetwork are connected to the other proteins of the directed osteoclast differentiation network. [file peerj-06-5743-s019.png]

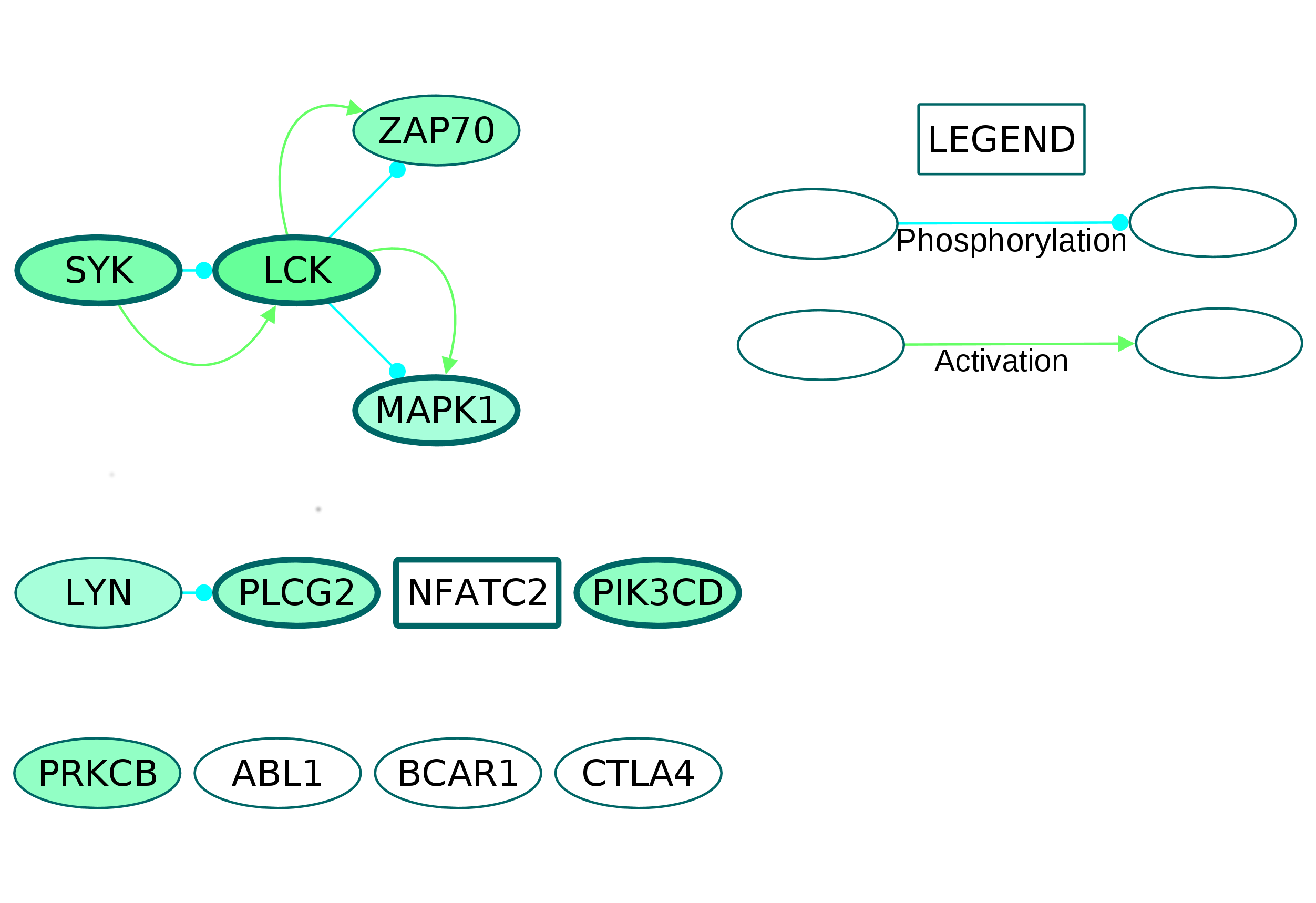

Supplement: Supplemental Information 20 — The core proteins are represented by thick borders and the shell proteins by thin borders. Rectangle nodes represent the DNA-binding proteins. The RA drug targets are indicated by red borders. The degree of differential regulation of the nodes is denoted as follows: red to grey–downregulation and green–upregulation. The proteins lying isolated in the subnetwork are connected to the other proteins of the directed osteoclast differentiation network. [file peerj-06-5743-s020.png]

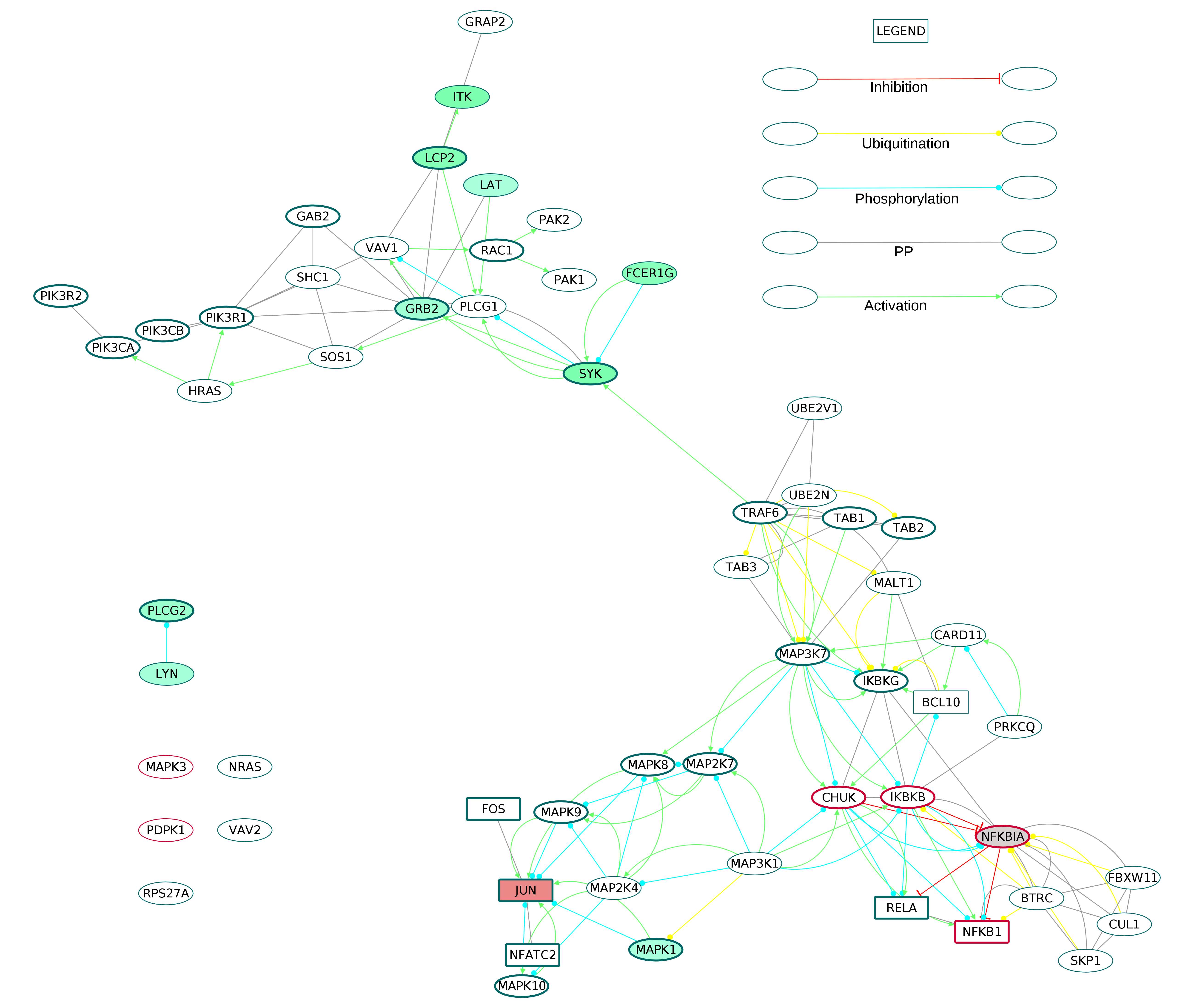

Supplement: Supplemental Information 21 — The core proteins are represented by thick borders and the shell proteins by thin borders. Rectangle nodes represent the DNA-binding proteins. The RA drug targets are indicated by red borders. The degree of differential regulation of the nodes is denoted as follows: red to grey–downregulation and green–upregulation. The proteins lying isolated in the subnetwork are connected to the other proteins of the directed osteoclast differentiation network. [file peerj-06-5743-s021.png]

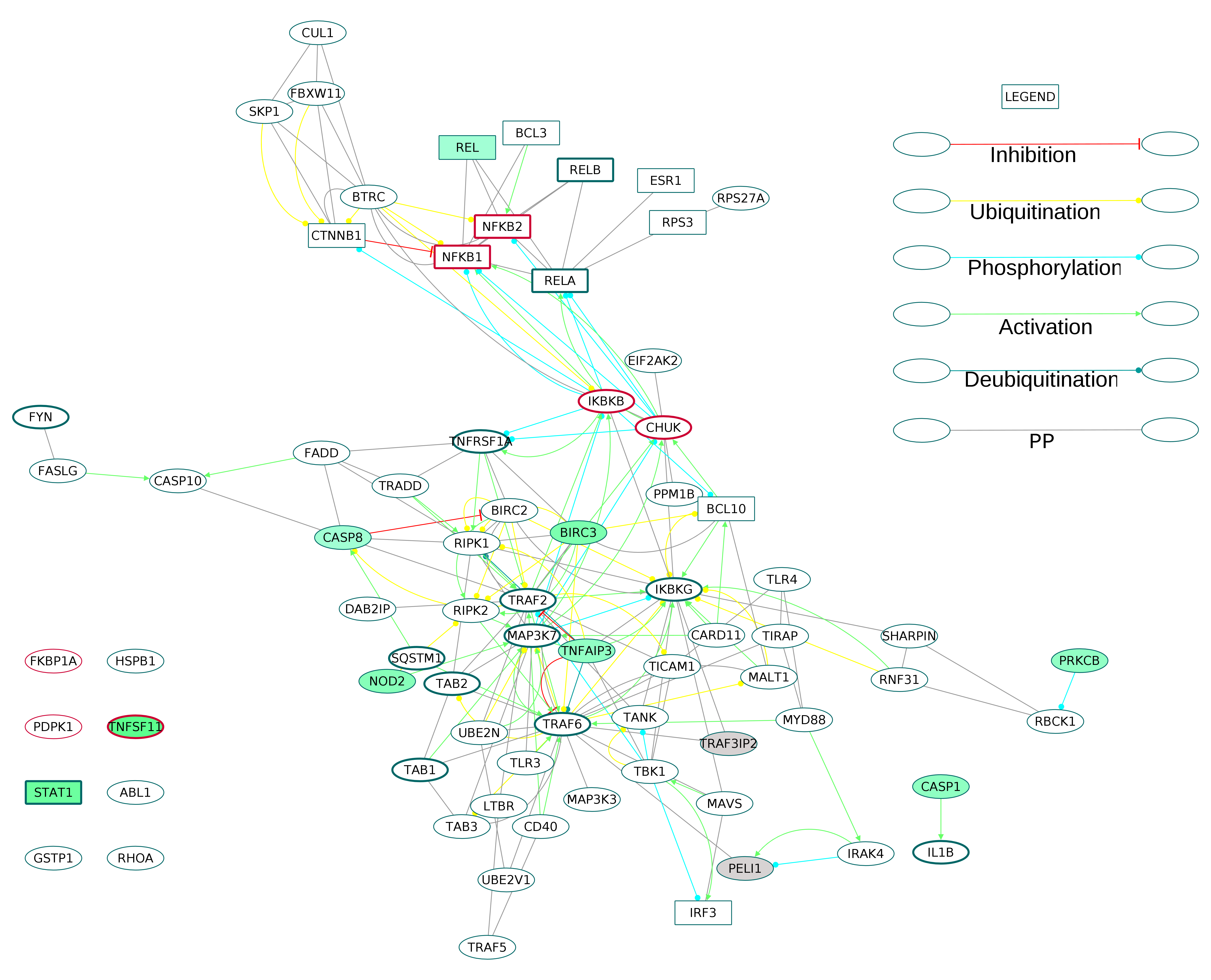

Supplement: Supplemental Information 22 — The core proteins are represented by thick borders and the shell proteins by thin borders. Rectangle nodes represent the DNA-binding proteins. The RA drug targets are indicated by red borders. The degree of differential regulation of the nodes is denoted as follows: red to grey–downregulation and green–upregulation. The proteins lying isolated in the subnetwork are connected to the other proteins of the directed osteoclast differentiation network. [file peerj-06-5743-s022.png]

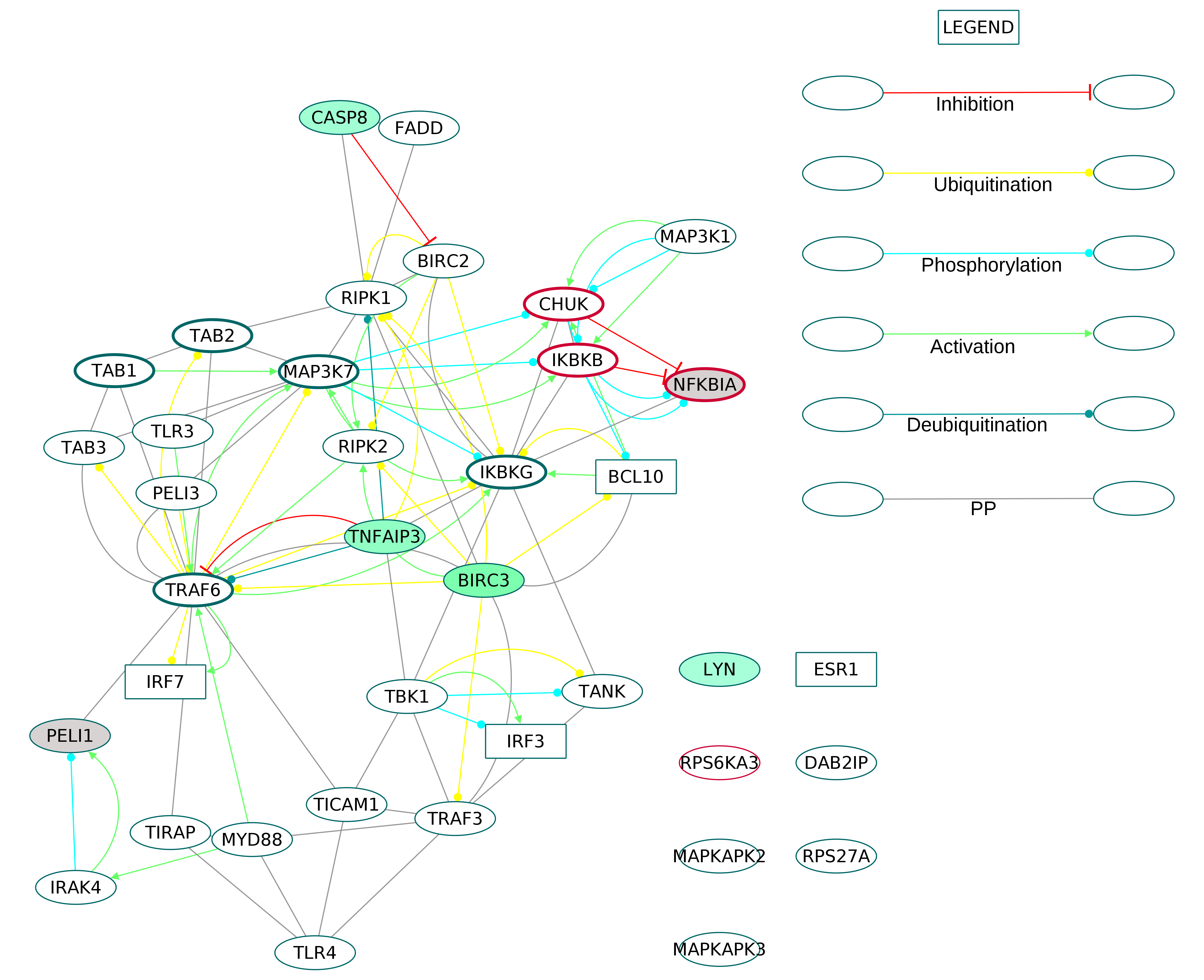

Supplement: Supplemental Information 23 — The core proteins are represented by thick borders and the shell proteins by thin borders. Rectangle nodes represent the DNA-binding proteins. The RA drug targets are indicated by red borders. The degree of differential regulation of the nodes is denoted as follows: red to grey–downregulation and green–upregulation. The proteins lying isolated in the subnetwork are connected to the other proteins of the directed osteoclast differentiation network. [file peerj-06-5743-s023.png]

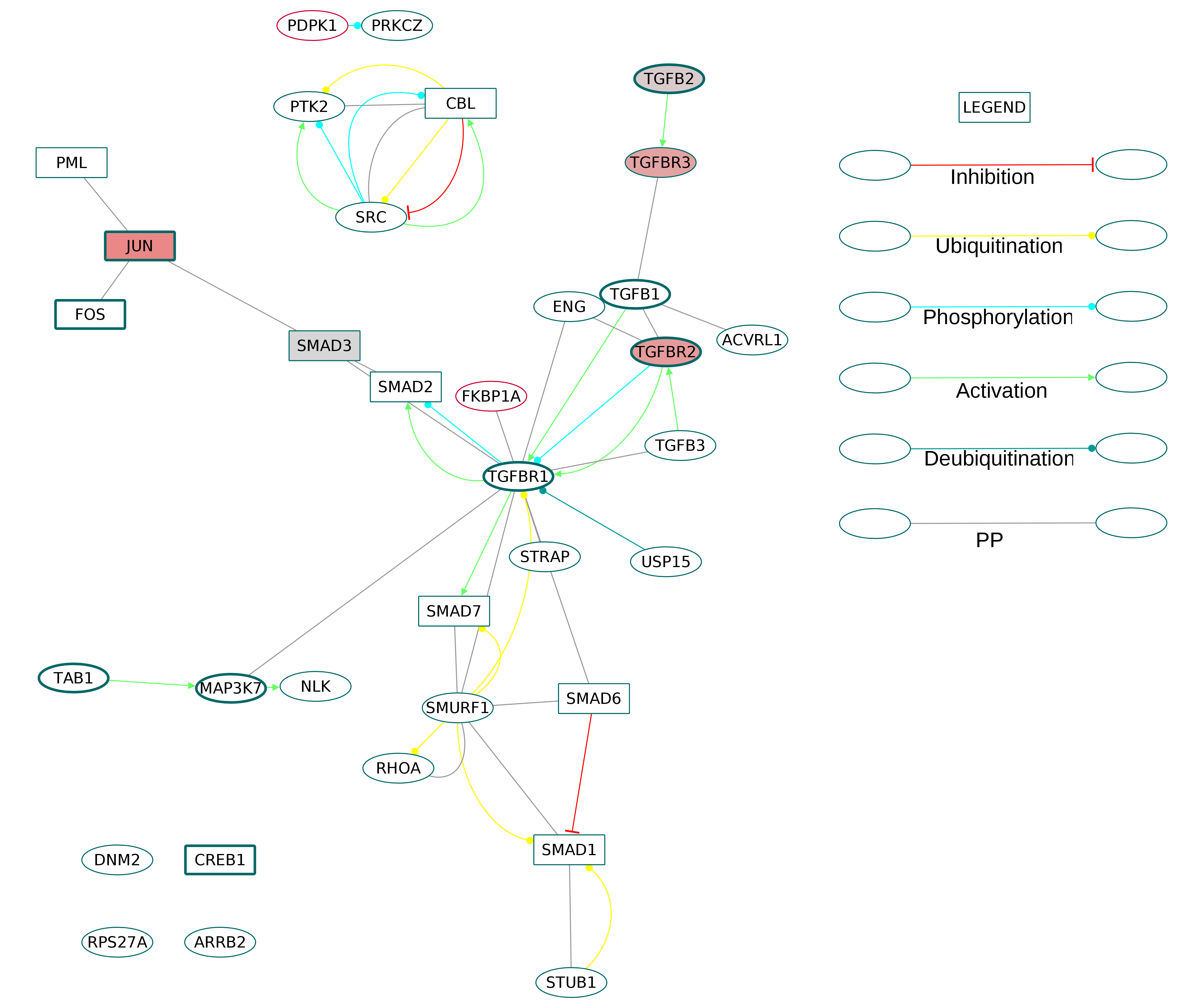

Supplement: Supplemental Information 24 — The core proteins are represented by thick borders and the shell proteins by thin borders. Rectangle nodes represent the DNA-binding proteins. The RA drug targets are indicated by the red borders. The degree of differential regulation of the nodes is denoted as follows: red to grey–downregulation and green–upregulation. The proteins lying isolated in the subnetwork are connected to the other proteins of the directed osteoclast differentiation network. [file peerj-06-5743-s024.png]
